# Emotional Intelligence, Self-Efficacy and Empathy as Predictors of Overall Self-Esteem in Nursing by Years of Experience

**DOI:** 10.3389/fpsyg.2019.02035

**Published:** 2019-09-18

**Authors:** María del Carmen Pérez-Fuentes, María del Mar Molero Jurado, Rosa María del Pino, José Jesús Gázquez Linares

**Affiliations:** ^1^Department of Psychology, Faculty of Psychology, University of Almería, Almería, Spain; ^2^Department of Psychology, Faculty of Psychology, Universidad Politécnica y Artística del Paraguay, Asunción, Paraguay; ^3^Department of Psychology, Faculty of Psychology, Universidad Autónoma de Chile, Santiago, Chile

**Keywords:** emotional intelligence, self-efficacy, empathy, self-esteem, nursing

## Abstract

Empirical research on self-esteem has a special interest in nursing professionals, because they work in a challenging environment that requires, in order to continue working during their working age, a strong physical, social and emotional involvement. The objective was to determine the explanatory value of individual variables such as Self-efficacy, Empathy and Emotional Intelligence on Self-esteem in a sample of nursing professionals, to identify which variables have the most explanatory value for overall self-esteem with years of experience. The study sample consisted of a total of 1,601 Spanish nurses aged 22–60, with a mean age of 31.19 (*SD* = 6.59) years. The emotional intelligence factors in all cases were observed to have positive correlations with overall self-esteem. Mood was still the predictor with the most explanatory weight in all the groups. General Self-Efficacy disappeared in the model of professionals with the most experience. Organizations and management nursing should implement intervention programs that promote wellbeing and satisfaction in workers during their working age. It would be of interest to train nursing professionals in such necessary competences as Empathy, Emotional Intelligence and Self-Efficacy.

## Introduction

The concept of self-esteem was originally introduced by James (1984) to refer to the extent to which individuals evaluate their own perceived success or failure in achieving their goals. In his book, “Principles of Psychology,” he wrote: “*So our self-feeling in this world depends entirely on what we back ourselves to be and do. It is determined by the ratio of our actualities to our supposed potentialities; a fraction of which our pretensions are the denominator and the numerator our success”* (James, 1890, p. 310).

Although there are numerous historical references, one of the authors who has contributed most to its study has been Moris Rosenberg, who defined it as “*a person's general feeling of worth*” (Rosenberg, 1965, p. 31).

Self-esteem begins developing in adolescence and evolves through middle age, arriving at its highest point at around 60 years of age, and starting at that age, slowly begins to decrease (Orth et al., 2012). Although the level of self-esteem may fluctuate throughout life in response to concrete daily experiences, it usually remains stable over long periods of time (Webster et al., 2017).

Some longitudinal studies have shown that although the trajectory of development of self-esteem follows a curve in all individuals, there are personal differences in the development of its various dimensions, that is different self-evaluations in specific facets (e.g., personal, social, academic, physical) (Orth, 2017). Thus, high self-esteem is associated with wellbeing and satisfaction (Orth et al., 2012), psychological adjustment (Liu et al., 2014), socialization and social inclusion (Orth, 2017), coping with conflictive situations and managing stress effectively (Bajaj et al., 2016; Yildirim et al., 2017), and positive organizational results (Xanthopoulou et al., 2007). While individuals with low self-esteem tend to have a negative view of the world (Cameron and Granger, 2018), problems with health and psychological disorders (e.g., depression; Michalak et al., 2011), they are, in addition, at risk of burnout (Alharbi et al., 2016; Molero et al., 2018; Pérez-Fuentes et al., 2019).

In view of the above, there is considerable interest in its study in professional and academic fields due to the wide range of repercussions affecting various personal domains (e.g., social, health, work). Empirical research on self-esteem has a special interest in nursing professionals, because they work in a challenging environment which requires strong physical, social and emotional involvement (Edwards et al., 2010). Similarly, positive personal evaluations influence personal resources, increasing wellbeing and satisfaction of employees and in the organization (e.g., engagement, better therapeutic relationship with patients) (Lehuluante et al., 2012; Lin et al., 2018; Pérez-Fuentes et al., 2018a).

Empirical studies on self-esteem have not only taken an interest in delving deeper into its evolution, changes, variations and implications; different lines of research have also emerged which explore the factors influencing this construct (Orth, 2017). For example, some studies have concentrated on determining the predictive capacity of Big Five Personality Traits on self-esteem (Weidmann et al., 2017; Pilarska, 2018). Another influential branch of research directly examined how certain individual self-judgments influence self-esteem, among which perceived Self-Efficacy, Empathy and Emotional Intelligence (EI) are emphasized.

Perceived Self-efficacy refers to an individual's belief, derived from his or her own experiences, about their ability to control their surroundings (Bandura, 1977). It is a personal construct widely studied in the scope of Organizational Psychology, and is considered a powerful antecedent for engagement and job performance, as well as a buffer against burnout (Barbaranelli et al., 2018) and is taken into account in instruments that value the burnout from a multidimensional perspective (Pérez-Fuentes et al., 2017, 2018b). On the other hand, longitudinal studies have demonstrated that positive thoughts about one's Self-efficacy contribute to maintaining optimal levels of self-esteem to the extent that individuals feel they have a greater ability to confront stressful situations (Caprara et al., 2013). Thus, some authors have found a positive relationship between Self-Efficacy and self-esteem (Maggiori et al., 2016).

Empathy is “*an individual's ability to understand and share the feelings of others*” (Jolliffe and Farrington, 2006, p. 589). It consists of two dimensions, Affective Empathy, which involves an emotional response and Cognitive Empathy, which consists of a rational understanding of the emotions (Villadangos et al., 2016; Slavny and Moore, 2018). In the field of healthcare, it is considered an essential quality for professionals in relating to patients (Petrucci et al., 2016). Thus, individuals who react empathically to others tend to increase their feeling of self-esteem (Cameron and Fredrickson, 2015). Although empathy is a major component of healthcare work, excessive identification with the emotions of patients and family members can generate anxiety and exhaustion in the professional (Kompanje et al., 2015; Schwan, 2018). Thus, increasing cognitive empathy, and thereby, diminishing the affective component, generates benefits in the care, dedication and effectiveness of healthcare workers (Navarro-Abal et al., 2018; Shao et al., 2018).

Finally, Emotional Intelligence (EI) consists of those skills a person possesses for understanding, perceiving and adaptively regulating their own emotions and those of others (Salovey and Mayer, 1990; Nightingale et al., 2018). According to the model proposed by Mayer and Salovey (1997), EI is defined as an independent cognitive ability associated with general intelligence. It consists of four different skills: perception of emotion, emotional facilitation, understanding emotions and their management. Meanwhile, Bar-On (1997) described a mixed model of IE consisting of different emotional and social skills grouped into five interacting areas: Intrapersonal (including emotional awareness, assertiveness, independence, self-esteem and self-realization); Interpersonal (including empathy, social responsibility and interpersonal relationships); Stress management (including stress tolerance and impulse control); and Mood (including happiness and optimism). Baron's concept of EI (2006) is based on the ability to understand oneself and others, cope with environmental demands and solve everyday problems in changing situations.

After considering both of the above EI models, EI involvement in the performance of nursing professionals, and its usefulness for their interaction with colleagues, patients and family members, we decided on the Bar-On model as a theoretical basis for quantifying the IE construct. Furthermore, such mixed models attempt to understand how the ability to control stress affects the ability to concentrate and use intelligence, for example, in decision-making. In nursing, decision-making processes could be said to be a constant, associated with self-efficacy, and ultimately, with the professional's self-esteem.

Several studies have revealed that EI is related to a positive emotional state and high self-esteem (Carvalho et al., 2018). The relationship between dimensions of EI and self-esteem, positiveness and optimism (“Mood,” Bar-On, 2006), for example, which strengthen self-concept by effectively managing stressful situations, has also been explored (Mäkikangas et al., 2004); adequate coping strategies and conflict resolution (“Adaptability”; Bar-On, 2006) are related to good self-esteem (Yildirim et al., 2017); the ability to be aware of one's own thoughts and feelings (“Intrapersonal”; Bar-On, 2006) keeps individuals from becoming absorbed in negative or critical beliefs, thereby improving their self-esteem (Bajaj et al., 2016); likewise, emotional regulation helps counteract the negative impact of stressful situations (“Stress management”; Bar-On, 2006), promoting positive affect in emotional self-evaluation (Park and Dhandra, 2017).

Finally, it has been shown that work experience, that is, years of seniority in the profession affects both satisfaction of workers (Lehuluante et al., 2012) and the way they behave in and cope with certain situations (Chang et al., 2016); Pasila et al. (2017) also found that nurses with little work experience are at a high risk of leaving the profession due to work stress.

Our objective was to determine the explanatory value of individual variables such as Self-efficacy, Empathy and Emotional Intelligence on Self-esteem in a sample of nursing professionals, and further, to identify which variables have the most explanatory value for overall self-esteem with years of experience.

## Methods

### Participants

The study sample consisted of a total of 1,601 Spanish nurses aged 22–60, with a mean age of 31.19 (*SD* = 6.59) years. Of the total sample, 15.3% (*n* = 245) were men and 84.7% (*n* = 1,356) women, with mean ages of 31.18 (*SD* = 6.77) and 31.19 years, respectively (*SD* = 6.56). The sample had a mean of 6.32 (*SD* = 5.87) years of experience in the profession in a range of 0 to 35 years. Classification of the variable also led to four groups with the following distribution: Group 0 (none or less than 1 year experience) 16.5% (*n* = 264), Group 1 (1–4 years) 31.5% (*n* = 505); Group 2 (5–10 years) 29.5% (*n* = 473); and Group 3 (11 years or over) 22.4% (*n* = 359).

### Instruments

To collect sociodemographic (age, sex) and professional data (years' experience in the profession), an *ad hoc* questionnaire was used.

Rosenberg Self-Esteem Scale (Rosenberg, 1965). This ten-item instrument for evaluating self-esteem was originally developed for its application to an adolescent population. It evaluates self-acceptance and self-respect. The subjects must answer on a four-choice Likert-type scale, where the extremes in the response range are in “strongly agree” and “strongly disagree.” This scale has shown adequate psychometric characteristics, in this case, the scale's alpha was 0.86.

General Self-Efficacy Scale (Baessler and Schwarcer, 1996). Based on 10 items, this scale evaluates the degree to which subjects perceive themselves to be generally competent in the effective management of stressful situations. The reliability analysis done with the study sample resulted in an alpha of 0.92, somewhat higher than the one found by other authors (0.87) with a university population (Sanjuán et al., 2000) or with adolescents (Verdugo et al., 2018).

Brief Inventory of Emotional Intelligence for Senior Citizens (EQ-i-20M; Pérez-Fuentes et al., 2014). This is a Spanish adaptation for an adult Spanish population of the Emotional Intelligence Inventory: Young Version (EQ-i-YV) proposed by Bar-On and Parker (2000). The inventory measures five emotional intelligence dimensions: Intrapersonal, Interpersonal, Stress management, Adaptability and Mood. Based on 20 items in which the subject must answer on a four-point Likert-type response scale. With this study sample, the Cronbach's alpha for the total scale was 0.89, and for the subscales from 0.76 to 0.91.

Basic Empathy Scale (BES; Jolliffe and Farrington, 2006). Specifically, for this study, the Spanish adaptation was used (Oliva et al., 2011) was used. It evaluates empathy based on two dimensions differentiating affective and cognitive empathy. Answers are given on a five-choice response scale, in which subjects could place their response on a continuum from “strongly disagree” to “strongly agree.” The total score for each of the dimensions is found from the sum of the items, so that the higher the score the more intense the empathic attitude is. Oliva et al. (2011) found the scale's internal consistency to be 0.73 for affective empathy and 0.63 for cognitive empathy. In our case, the analyses of internal consistency in the study sample showed an alpha of 0.86 on both scales.

### Procedure

First, the participants were informed of the objectives of the research and guaranteed anonymity of their answers and compliance with the ethical standards of confidentiality in data processing. The study was approved by the Bioethics Committee of the University of Almeria (Ref: UALBIO2017/011). The questionnaires used for data collection were implemented on a Web platform in 2017, which enabled them to be filled out online. In order to avoid random or incongruent answers, control questions were inserted to enable any anomalies or biases in the answers to be identified. In addition to these cases, incomplete questionnaires were also discarded.

### Data Analysis

#### Cross-Sectional Study

First, to identify the relationships of the individual variables (self-efficacy, empathy and emotional intelligence) with overall self-esteem, in addition to the corresponding descriptive statistics, the Pearson's correlation coefficient was calculated. Then a stepwise multiple linear regression analysis was done for the total sample and for each of the groups of professionals by years of experience to find out how the predictor variables (General self-efficacy, Affective and Cognitive Empathy, Intrapersonal and Interpersonal Emotional Intelligence, Stress Management, Adaptability and Mood) were related to the criterion variable. For this analysis, the variable “years of experience” was classified by visual grouping and by percentiles based on the cases explored. Taking one year's experience as the starting point, two cutoff points were taken (33.3%), creating three intervals with approximately the same number of cases in each category. Groups were formed by years of experience. First, the participants who had had less than 1 year of experience were separated from the rest. The cut-off points were identified by visual grouping, and finally the variable was recoded manually.

## Results

### Overall Self-Esteem and Its Relationship With General Self-Efficacy, Empathy and Emotional Intelligence

The descriptive statistics of each of the variables in the study are shown in Table 1. The correlation coefficients reveal that professionals with high self-efficacy also had higher scores in overall self-esteem (*r* = 0.52; *p* < 0.001). Affective empathy (*r* = −0.09; *p* < 0.001) had a negative correlation with self-esteem and cognitive empathy (*r* = 0.16; *p* < 0.001) a positive correlation.

**Table 1:** Descriptive statistics and correlations for overall self-esteem, general self-efficacy, empathy, and emotional intelligence (*N* = 1601).

	**AESover**	**AEFgen**	**Empathy**		**Emotional intelligence**				
			**EMPa**	**EMPc**	**IEintra**	**IEinter**	**IEsm**	**IEadap**	**IEm**
Mean	26.00	31.40	14.36	19.91	10.59	12.17	12.92	11.42	12.39
*SD*	3.80	4.56	2.94	2.62	2.68	1.85	2.00	2.07	2.20
AEFgen	0.52^***^	–							
EMPa	−0.09^***^	−0.04	–						
EMPc	0.16^***^	0.38^***^	0.36^***^	–					
IEintra	0.39^***^	0.35^***^	0.03	0.27^***^	–				
IEinter	0.30^***^	0.44^***^	0.14^***^	0.56^***^	0.40^***^	–			
IEsm	0.26^***^	0.21^***^	−0.09^***^	0.09^***^	0.14^***^	0.12^***^	–		
IEadap	0.47^***^	0.64^***^	−0.07^**^	0.33^***^	0.43^***^	0.57^***^	0.20^***^	–	
IEm	0.71^***^	0.60^***^	−0.10^***^	0.24^***^	0.46^***^	0.40^***^	0.28^***^	0.58^***^	–

*AESglob, Overall Self-esteem; AEFgen, General Self-Efficacy; EMPa, Affective empathy; EMPc, Cognitive empathy; IEintra, Intrapersonal; IEinter, Interpersonal; IEsm, Stress management; IEadap, Adaptability; IEm, Mood*.

** *p < 0.01*,

*** *p < 0.001*.

The emotional intelligence factors in all cases were observed to have positive correlations with overall self-esteem (Intrapersonal: *r* = 0.39; *p* < 0.001; Interpersonal: *r* = 0.30; *p* < 0.001; Stress management: *r* = 0.26; *p* < 0.001; Adaptability: *r* = 0.47; *p* < 0.001; Mood: *r* = 0.71; *p* < 0.001).

### Overall Self-Esteem Predictors in Nursing. Total Sample

According to the data shown in Table 2, the regression analysis resulted in five models, the last of which is the one with the highest explanatory capacity, with 53.1% (*R*^2^ = 0.53) of the variance explained by the factors included in the model.

Table 2Stepwise multiple linear regression model (total sample: *N* = 1601).

**Table:** 

**Model**	***R***	***R*^**2**^**	***R*^**2**^ corrected**	**Change statistics**				**Durbin Watson**
				**Standard error of estimation**	**Change in *R*^**2**^**	**Change in *F***	**Sig. of change in *F***	
1	0.71	0.51	0.51	2.66	0.51	1665.11	0.000	1.92
2	0.72	0.52	0.52	2.63	0.01	41.71	0.000	
3	0.72	0.52	0.52	2.62	0.00	10.15	0.001	
4	0.72	0.52	0.52	2.61	0.00	8.70	0.003	
5	0.72	0.53	0.52	2.61	0.00	8.98	0.003

**Table:** 

**Model 5**	**Non-standardized coefficients**		**Standardized coefficients**	***t***	**Sig**.	**Collinearity**	
	***B***	**Std. Error**	**Beta**			**Tol**.	**VIF**
(Constant)	8.70	0.66		13.14	0.000		
Mood	1.01	0.04	0.59	25.29	0.000	0.54	1.85
General Self-efficacy	0.012	0.01	0.14	6.57	0.000	0.57	1.74
Stress management	0.10	0.03	0.05	3.19	0.001	0.91	1.09
Intrapersonal	0.09	0.02	0.06	3.40	0.001	0.75	1.33
Cognitive empathy	−0.08	0.02	−0.05	−2.99	0.003	0.83	1.20

Residual independence was analyzed to confirm the model's validity. The Durbin-Watson D was *D* = 1.92, confirming absence of positive and negative self-correlation. It was further observed that the *t* was associated with a probability of error under 0.05 in all variables included in the model (Mood, General Self-Efficacy, Stress Management, Intrapersonal and Cognitive Empathy). The standardized coefficients reveal that the variable with the most explanatory weight is Mood. Finally, based on the values found for the tolerance and VIF indicators, absence of collinearity of the variables included in the model may be assumed.

### Predictors of Overall Self-Esteem in Nursing by Years of Experience in the Profession

Table 3 shows the models that resulted from the multiple linear regression analysis, taking years of experience in the profession as the selection variable and entering the category which divides the sample into four groups (Group 0 = no experience or less than one year; Group 1 = 1–4 years; Group 2 = 5–10 years; and Group 3 = 11 years or over) in each case, as the selection criterion.

Table 3Stepwise multiple linear regression model, by years of professional experience (Group 0: *n* = 264; Group 1: *n* = 505; Group 2: *n* = 473; Group 3: *n* = 359).

**Table:** 

	**Model**	***R***	***R*^**2**^**	**Corrected *R*^**2**^**	**Change statistics**				**Durbin Watson**
					**Standard error of estimation**	**Change in *R*^**2**^**	**Change in *F***	**Sig. of change in *F***	
GROUP 0 (<1 year)	1	0.72	0.52	0.52	2.61	0.52	288.01	0.000	1.83
	2	0.73	0.53	0.53	2.59	0.01	5.81	0.017	
	3	0.73	0.54	0.53	2.57	0.00	4.21	0.041

**Table:** 

	**Model 3**	**Non-standardized coefficients**		**Standardized coefficients**			**Collinearity**	
		***B***	**Standard error**	**Beta**	***t***	**Sig**.	**Tol**.	**VIF**
	(Constant)	10.56	1.60		6.57	0.000		
	Mood	1.24	0.07	0.73	16.44	0.000	0.88	1.12
	General self-efficacy	0.19	0.08	0.10	2.36	0.019	0.97	1.03
	Cognitive empathy	−0.12	0.06	−0.09	−2.05	0.041	0.91	1.09

**Table:** 

	**Model**	***R***	***R***^**2**^	**Corrected** ***R***^**2**^	**Change statistics**				**Durbin Watson**
					**Standard error of estimation**	**Change in** ***R***^**2**^	**Change in** ***F***	**Sig. of change in** ***F***	
GROUP 1 (1-4 years)	1	0.72	0.52	0.52	2.68	0.52	554.43	0.000	2.01
	2	0.73	0.53	0.53	2.64	0.01	15.25	0.000

**Table:** 

	**Model 2**	**Non-standardized coefficients**		**Standardized coefficients**	***t***	**Sig**.	**Collinearity**	
		***B***	**Standard error**	**Beta**			**Tol**.	**VIF**
	(Constant)	8.05	0.86		9.27	0.000		
	Mood	1.10	0.06	0.63	16.74	0.000	0.63	1.56
	General Self-efficacy	0.13	0.03	0.14	3.90	0.000	0.63	1.56

**Table:** 

	**Model**	***R***	***R***^**2**^	**Corrected** ***R***^**2**^	**Change statistics**				**Durbin Watson**
					**Standard error of estimation**	**Change in** ***R***^**2**^	**Change in** ***F***	**Sig. of change in** ***F***	
GROUP 2 (5-10 years)	1	0.71	0.51	0.51	2.61	0.51	496.80	0.000	2.13
	2	0.73	0.53	0.53	2.56	0.02	21.42	0.000

**Table:** 

	**Model 2**	**Non-standardized coefficients**		**Standardized coefficients**	***t***	**Sig**.	**Collinearity**	
		***B***	**Standard error**	**Beta**			**Tol**.	**VIF**
	(Constant)	8.29	0.82		10.06	0.000		
	Mood	1.05	0.07	0.59	14.72	0.000	0.60	1.66
	General Self-efficacy	0.14	0.03	0.18	4.62	0.000	0.60	1.66

**Table:** 

	**Model**	***R***	***R***^**2**^	**Corrected** ***R***^**2**^	**Change statistics**				**Durbin Watson**
					**Standard error of estimation**	**Change in** ***R***^**2**^	**Change in** ***F***	**Sig. of change in** ***F***	
GROUP 3 (≥11 years)	1	0.69	0.48	0.47	2.72	0.48	329.94	0.000	1.91
	2	0.70	0.50	0.49	2.67	0.02	14.11	0.000	
	3	0.71	0.51	0.50	2.64	0.01	7.66	0.006

**Table:** 

	**Model 3**	**Non-standardized coefficients**		**Standardized coefficients**	***t***	**Sig**.	**Collinearity**	
		***B***	**Standard error**	**Beta**			**Tol**.	**VIF**
	(Constant)	10.19	0.87		11.69	0.000		
	Mood	0.92	0.08	0.55	11.33	0.000	0.58	1.70
	Adaptability	0.24	0.08	0.13	2.83	0.005	0.57	1.74
	Intrapersonal	0.18	0.06	0.12	2.76	0.006	0.70	1.41

First, focusing on the group of professionals with the least experience (<1 year), three regression models were found, the last of which showed an explained variance of 54.1% (*R*^2^ = 0.54). The validity of the model determined with the Durbin-Watson *D* was near 2 (*D* = 1.83). The association of the variables showed a probability below 0.05 for Mood, Stress Management and Cognitive Empathy, which in this case, were the variables included in the model. According to the standardized coefficients, Mood was the variable with the highest explanatory value. Absence of collinearity of variables was also assumed according to the high tolerance and low VIF indicators.

In the group of professionals who had 1–4 years of experience, the regression analysis found two models. In the second, the variables in the equation were Mood and General Self-Efficacy, as the coefficients satisfying the condition of association with a value below 0.05. In this case, the percentage of variance explained by the second model was 53.8% (*R*^2^ = 0.53) and *D* = 2.01, confirming its validity. Furthermore, the tolerance and VIF indicators enabled absence of collinearity to be assumed.

In addition, in the group of participants with 5–10 years' experience, two models were found with the regression analysis, the second of which showed a higher percentage of explained variance of 53.5% (*R*^2^ = 0.53). Model validity was determined by the Durbin-Watson *D* as *D* = 2.13. The association of the variables showed a probability of less than 0.05 for both Mood and General Self-Efficacy, the variables included in the model. Of the two, Mood was the variable which had the most explanatory value. Furthermore, absence of collinearity of the variables included in the model was assumed based on the tolerance and VIF indicator values.

Finally, three models resulted from the multiple regression analysis for the group with the most years of experience in the profession (≥11 years). Three of the emotional intelligence dimensions were included in the third model, Mood, Adaptability and Intrapersonal, with an explained variance of 51.1% (*R*^2^ = 0.51). To confirm the model's validity, residual independence was analyzed using the Durbin-Watson *D*, which was *D* = 1.91, confirming the absence of positive or negative self-correlation. Furthermore, the *t* was associated with a probability of error below 0.05 in the variables in the model. The standardized coefficients revealed that the variable showing the most explanatory value in the equation was Mood. Based on the tolerance and VIF indicators, absence of collinearity of the variables in the model was assumed.

## Discussion

Since the beginning of the twentieth century, special scientific interest has been awakening in the study of self-esteem in nursing professionals as a group with high emotional, physical and social wear. However, a majority of the empirical research has concentrated on samples of students and nurses with little or no work experience (Edwards et al., 2010). Thus, one of the strengths of this study is its sample, which includes nurses with up to over 11 years of experience in the profession.

In parallel to the evidence already shown in previous studies (Maggiori et al., 2016), Self-Efficacy, Cognitive Empathy and all the dimensions of EI correlated positively with Self-Esteem. On the contrary, a negative relationship was found between Affective Empathy and Self-Esteem, which may be explained by emotional exhaustion. In fact, others authors have shown that emotional involvement, characteristic of Affective Empathy, impedes healthcare professionals from working effectively. While the ability to empathize based on understanding the patient's emotions has positive effects on the healthcare professional's response (Navarro-Abal et al., 2018; Shao et al., 2018), the affective component of empathy can be a disadvantage for clinical practice, since it generates feelings of excessive responsibility and insecurity about caregiving and patient care (Schwan, 2018). This negatively affects the feeling of self-efficacy, and ultimately, the self-esteem of the healthcare professional. Along this line, the negative repercussion of strong affective empathy on the effectiveness of nursing work could be explained by excessive emotional involvement that leads to burnout (Kompanje et al., 2015).

The results of our study for the total sample of nurses, confirmed previous studies which identified General Self-Efficacy (Caprara et al., 2013), Cognitive Empathy (Cameron and Fredrickson, 2015), and three Emotional Intelligence dimensions, Mood, Stress Management and Intrapersonal (Carvalho et al., 2018) as predictors of self-esteem. However, it should be emphasized that Mood is the most important predictor. Similarly, Mäkikangas et al. (2004) showed that positiveness and optimism favor interpretation of ambiguous or potentially stressful situations as positive, making individuals strengthen their self-concept about their ability to overcome adverse situations. Thus, Mood, as a component of Emotional Intelligence that emphasizes positive attitude and disposition as a coping strategy in stressful situations, is one of the most important determining factors in the self-esteem model proposed. These results also show the need to check whether self-efficacy mediates between Mood and self-esteem. This is an interesting proposal for future research.

Differences were found with years of experience for the variables which best predict self-esteem. Mood was still the predictor with the most explanatory weight in all the groups. Thus, nursing professionals with more seniority usually act more appropriately when faced with unexpected and very stressful situations (e.g., patient death) (Adaptability) (Chang et al., 2016). They also usually think more positively and feel greater satisfaction when performing their jobs (Intrapersonal) in comparison with those who have been working fewer years (Lehuluante et al., 2012; Pasila et al., 2017). The General Self-Efficacy disappeared in the model of professionals with the most experience. Cognitive empathy, however, only appears as a “negative” predictor for the group of nurses with the least experience. Even though studies have shown that self-esteem is an essential ingredient in healthcare professionals (Petrucci et al., 2016), it is true that for some employees with little experience it is emotionally very draining to understand the emotional states of other persons, which may be due to lack of training and/or preparation in this skill.

Nevertheless, this study could pose some limitations. First, the data may show bias from having been acquired with self-report questionnaires, although this is common in psychological evaluation. Finally, the study design did not allow it to be determined whether the relationships between Self-efficacy, Empathy and Emotional Intelligence and Self-Esteem are maintained over time, so it would be necessary to carry out longitudinal studies which make it possible to analyze the influence of the variables on self-esteem.

## Conclusions

A negative relationship was found between Affective Empathy and Self-Esteem which may be explained by emotional exhaustion, it should be emphasized that Mood is the most important predictor. Mood was still the predictor with the most explanatory weight in all the groups with years of experience. However, General Self-Efficacy disappeared in the model of professionals with the most experience, in which case two variables entered in the equation which were not present in the rest of the groups, Adaptability and Intrapersonal. Therefore, in view of the results, it might be concluded that depending on seniority in the position, professionals depend less on self-evaluation of their performance (Perceived Self-efficacy) and more on their Emotional Intelligence (Mood, Adaptability, Intrapersonal) to configure their self-esteem.

The evidence which has emerged from this study may have important practical implications. In the academic sphere, it would be of interest to train future nursing professionals in such necessary competences as Empathy, Emotional Intelligence and Self-Efficacy. Similarly, organizations and nursing management should implement intervention programs that promote wellbeing and satisfaction in workers during their period of active employment. It would be of interest to train nursing professionals in such necessary competences as Empathy, Emotional Intelligence and Self-Efficacy. For example, by training in emotional management or improving response to patients and family members based on empathic communication.

For intervention, other ways of developing belief in self-efficacy should be developed. In line with the theory of Bandura (1994), one of the most effective ways to do this would be through experience, vicarious learning by observing social models, or by controlling stress, which is important for designing intervention aimed at reducing stress to control negative emotional reactions and their consequences, such as harmful somatization.

As future lines of research, it would be of interest to widen the set of variables used in this study, including aspects related to the demands (e.g., work overload, shifts) and resources (e.g., autonomy, leadership), as well as personality variables, for example, making use of the Big Five Personality Traits.

## Data Availability

The datasets generated for this study are available on request to the corresponding author.

## Ethics Statement

The participants were informed of the objectives of the research and guaranteed anonymity of their answers and compliance with the ethical standards of confidentiality in data processing. This study was approved by the Bioethics Committee of the University of Almeria (Ref: UALBIO2017/011).

## Author Contributions

MM and MP-F contributed to the conception and design of the review. JG applied the search strategy. MM, RP, and MP-F wrote this manuscript. MP-F and JG edited this manuscript. MP-F is responsible for the overall project. All authors applied the selection criteria, completed the assessment of risk of bias, and analyzed the data and interpreted data.

### Conflict of Interest Statement

The authors declare that the research was conducted in the absence of any commercial or financial relationships that could be construed as a potential conflict of interest. The reviewer AG-G declared a shared affiliation, with no collaboration, with the authors to the handling editor at time of review.
